# Motivation by reward jointly improves speed and accuracy, whereas task-relevance and meaningful images do not

**DOI:** 10.3758/s13414-022-02587-z

**Published:** 2022-10-26

**Authors:** Christian Wolf, Markus Lappe

**Affiliations:** Institute for Psychology, University of Münster, Fliednerstrasse 21, 48149 Münster, Germany

**Keywords:** Eye movements and visual attention, Eye movements: Cognitive, Reponse time models

## Abstract

Visual selection is characterized by a trade-off between speed and accuracy. Speed or accuracy of the selection process can be affected by higher level factors—for example, expecting a reward, obtaining task-relevant information, or seeing an intrinsically relevant target. Recently, motivation by reward has been shown to simultaneously increase speed and accuracy, thus going beyond the speed–accuracy-trade-off. Here, we compared the motivating abilities of monetary reward, task-relevance, and image content to simultaneously increase speed and accuracy. We used a saccadic distraction task that required suppressing a distractor and selecting a target. Across different blocks successful target selection was followed either by (i) a monetary reward, (ii) obtaining task-relevant information, or (iii) seeing the face of a famous person. Each block additionally contained the same number of irrelevant trials lacking these consequences, and participants were informed about the upcoming trial type. We found that postsaccadic vision of a face affected neither speed nor accuracy, suggesting that image content does not affect visual selection via motivational mechanisms. Task relevance increased speed but decreased selection accuracy, an observation compatible with a classical speed–accuracy trade-off. Motivation by reward, however, simultaneously increased response speed and accuracy. Saccades in all conditions deviated away from the distractor, suggesting that the distractor was suppressed, and this deviation was strongest in the reward block. Drift-diffusion modelling revealed that task-relevance affected behavior by affecting decision thresholds, whereas motivation by reward additionally increased the rate of information uptake. The present findings thus show that the three consequences differ in their motivational abilities.

## Introduction

Visual decision-making is characterized by a trade-off between speed and accuracy. When performing a visual selection task, we can prioritize accuracy, in which case our responses will most likely be slow. Conversely, if we prioritize speed, our behavior is sometimes premature and performance will be prone to errors. The speed–accuracy trade-off describes this lawful relationship between speed and accuracy for a fixed task difficulty (Fitts, 1954; Heitz, 2014; Standage et al., 2014). It can be found across a variety of tasks, for movements of different effectors (Harris & Wolpert, 2006; Michmizos & Krebs, 2014; Smyrnis et al., 2000) as well as across species (Chittka et al., 2003; Franks et al., 2009).

Speed–accuracy data are often modelled using sequential sampling models (for reviews see Bogacz et al., 2006; Heitz, 2014; Standage et al., 2014)—for example, the drift-diffusion model (Ratcliff, 1978; Ratcliff et al., 2016). Sequential sampling models commonly assume that information from the environment is constantly sampled until a threshold is reached and a response is carried out. A clear advantage of the drift-diffusion model and other sequential sampling models is that the joint modelling of speed and accuracy allows one to infer latent psychological variables like response bias, information uptake, and decision threshold (Voss et al., 2004). Whereas response bias can be affected by informing participants that one of the response options is more likely correct, information uptake reflects the participant’s ability to perform the task and can be affected by the stimulus quality. The latter is reflected in the drift rate parameter. Decision threshold on the other hand is reflected in the boundary separation parameter and can be affected when either speed or accuracy is emphasized. Changes in decision threshold are thus typically indicative of a speed–accuracy trade-off.

A recent study showed that motivation by monetary reward can operate outside the speed–accuracy trade-off by simultaneously speeding up responses and increasing response accuracy (Manohar et al., 2015). In that study, participants fixated one of three discs in a triangular arrangement. A recorded voice provided information about the maximum amount of reward that could be obtained in that trial. Then, the other two discs changed their luminance one after the other. The disc lit first was the distractor and had to be ignored, whereas the disc lit second was the target and had to be selected by means of a saccadic eye movement. Participants received monetary reward if they correctly selected the target disc. Importantly, the obtained reward decreased with increasing reaction time such that a mature response might only yield a small fraction of the announced maximum reward. The authors found that motivation by monetary reward decreased both reaction times and error rates, inconsistent with a speed–accuracy trade-off. The results were explained by a model that included a noise-reduction component that operates perpendicular to the speed–accuracy trade-off, but which comes at a cost. This precision cost can explain why motivation by reward can increase both speed and accuracy.

Earlier, faster or more accurate saccades have not only been reported in studies employing a monetary reward (Chen et al., 2014; Clark & Gilchrist, 2018; Dunne et al., 2015; Kojima & Soetedjo, 2017; Muhammed et al., 2020; Takikawa et al., 2002) but also when participants perform a perceptual task at the saccade target (Bieg et al., 2012; Guyader et al., 2010; Montagnini & Chelazzi, 2005; Schütz & Souto, 2015; Trottier & Pratt, 2005; Wolf & Schütz, 2017) or when selecting a particular image content for visual inspection—for example, a human face (Crouzet et al., 2010; Entzmann et al., 2021; Kauffmann et al., 2019; Meermeier et al., 2016, 2017; Xu-Wilson et al., 2009; for review, see Wolf & Lappe, 2021b). The latter findings support the view that foveal vision of a particular target can itself be rewarding and that this is reflected in eye-movement dynamics towards that target (Clark & Gilchrist, 2018; Collins, 2012; Shadmehr et al., 2010; Wolf & Lappe, 2021b). Yet a benefit in either speed or accuracy does not necessarily imply that obtaining task-relevant information or seeing an intrinsically relevant image affects selection processes via motivational mechanisms and, furthermore, that it can reduce internal noise and simultaneously increase speed and accuracy.

The aim of the present study was to test whether task-relevance and/or image content affect selection processes via motivational mechanisms, and second, whether task-relevance and image content simultaneously increase speed and accuracy. To test this, we adopted the paradigm introduced by Manohar et al. (2015) but varied the consequences following successful target selection. Across different blocks, participants either obtained a monetary reward, obtained task-relevant information for a perceptual task, or saw the face of a famous person. We compared speed and accuracy from these trials (relevant trials) with interleaved trials from the same block, lacking these consequences (irrelevant trials). At the beginning of each trial, participants were informed whether a trial was relevant (e.g., “reward,” “task,” “face”) or not. Importantly, peripheral targets were identical in all trials, excluding the possibility that performance was affected by low-level properties of peripheral targets which are known to affect eye movement characteristics (Crouzet & Thorpe, 2011; Honey et al., 2008; Itti & Koch, 2001). This enables attributing differences in speed and accuracy to motivational processes. We complement our analysis with a drift-diffusion modelling approach to attribute the observed differences in speed and accuracy to differences in decision threshold and information uptake.

## Methods

### Participants

We recorded data of 36 participants (mean age = 20 years, age range: 18–29, six males, 30 females). Participants were undergraduate students from the University of Münster and received course credit or 8€/h for participation. In addition, participants received a performance-dependent monetary reward of up to 9.60€ in the reward block (up to 0.10€/trial). Obtained rewards were rounded up to the first decimal after the comma and ranged from 2.60 to 7.00€ (median: 4.50€). Written informed consent was provided before testing. The experiment was approved by the ethics committee of the Department of Psychology and Sport Sciences of the University of Münster.

### Setup

Stimuli were presented on an Eizo FlexScan 22-inch CRT monitor (Eizo, Hakusan, Japan) with a resolution of 1,152 × 870 pixels, a refresh rate of 75 Hz, and an effective display size of 40.7 × 30.5 cm. Participants viewed stimuli from a 67 cm distance. Head movements were restricted by means of a chin–forehead rest. Stimulus presentation was controlled via the Psychtoolbox (Brainard, 1997; Kleiner et al., 2007) in MATLAB (The MathWorks, Natick, MA). Eye position of the right eye was recorded at 1000 Hz using the EyeLink 1000 (SR Research, Mississauga, ON, Canada) and the EyeLink Toolbox (Cornelissen et al., 2002). All stimuli were presented on a black background. The EyeLink was calibrated at the beginning of each block using a 9-point calibration protocol.

### Procedure and stimuli

The experiment comprised three blocks (reward, task, face). Each block contained 192 trials and consisted of two trial types that differed in terms of the consequences of a successful saccade. We refer to these two trial types as relevant and irrelevant trials. In relevant trials, participants either received a small monetary reward of up to 0.1€ (reward block), performed a perceptual orientation task at the saccade target (task block) or saw the face of a famous person (face block). In irrelevant trials, participants received no monetary reward (reward block), performed no perceptual task (task block), or saw a grating (face block). All blocks were recorded within one session of 60–90 minutes with breaks in-between blocks. The order of blocks was balanced across participants.

**Fig. 1:**
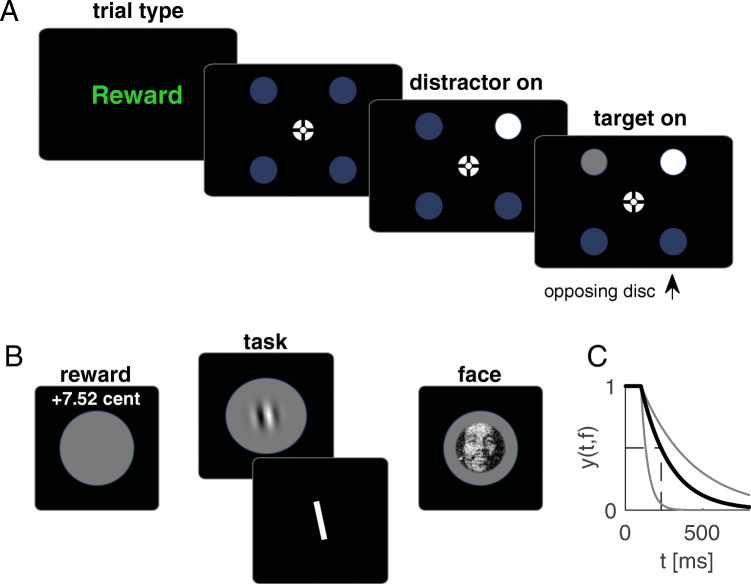
Trial procedure and critical manipulation. **a** Trial procedure common for all three blocks. A text at the beginning of each trial indicated whether the next trial was relevant (“reward,” “task,” “face”) or irrelevant (“no reward,” “no task,” “grating”). Four blue discs appeared, of which two changed successively. Participants had to look at the disc changing second (target) while ignoring the disc changing first (distractor). Given that distractor and target were always next to each other, knowing the distractor location renders two discs possible target locations: The actual target and the disc opposing the target (opposing disc). **b** Consequences following successful target selection in relevant trials of the three respective blocks. Participants either received a monetary reward, saw a face of a famous person, or had to perform a perceptual task at the saccade target. **c** Reward, image quality and tilt decayed with increasing reaction time. Thus, later responses came along with a diminished reward, worse image quality (and thus impaired recognition) or a more difficult perceptual task. The actual decay in each trial was derived from an exponential decay function. The black line denotes the decay function as it was set at the beginning of each block. The decay additionally depended on the median latency of the previous trials to assure a constant difficulty across the experiment and across participants. The initial decay corresponded to a median latency of 230 ms (dashed black lines). The thin gray lines show how the decay would have become steeper or shallower, if the median decreased or increased by 100 ms. (Color figure online)

At trial beginning, a centrally displayed text, shown for 1s (Fig. 1a), announced whether the trial was relevant (green font, “reward,” “task,” “face”) or irrelevant (red font, “no reward,” “no task,” “grating”). Afterwards, a central white fixation cross and four dark blue discs appeared. We used a combination of bull’s eye and hair cross as fixation marker (Thaler et al., 2013). Discs had a radius of 2 deg and were arranged in a 16-deg square pattern around the fixation point. Thus, the total distance between the fixation cross and each disc was approximately 11.3 deg. After a uniform random interval between 0.5 and 1 s, one of the four discs, the distractor, changed to white. After additional 187 ms, the target disc changed to gray. Target and distractor were either horizontally or vertically adjacent and were therefore always separated by 16 deg. Consequently, there were two potential target discs for every distractor location. A disc was labelled as selected by a gaze movement if gaze was less than 4 deg away from a disc center. All targets were removed 450 ms after disc selection or 760 ms after target onset. If no disc had been selected within 760 ms, the trial was labelled as too slow and repeated at the end of the experiment (<1% of trials). In that case a “too slow” message appeared at the screen center. Participants were instructed to look at the target. No instruction towards speed or accuracy was given.

### Consequences following target selection in the different blocks

Blocks differed with respect to the consequences following a successful saccade target selection (Fig. 1b). In the reward block, if the trial was relevant and participants selected the correct disc, they received a small monetary reward. The maximal reward in each trial was 0.1€ and decayed with increasing reaction time (see Decay, below). Feedback (e.g., “+7.52 cent”) was provided 0.8 deg above the selected disc as soon as the disc was selected. In irrelevant trials or if the wrong disc was selected “+0 cent” was displayed.

In the task block, a grating (Gabor patch) was displayed on the target disc upon selection. The grating had a spatial frequency of 1.69 cycles per degree and a Gaussian envelope with a standard deviation of 0.3 deg. The gratings orientation slightly deviated clockwise or counterclockwise from vertical. The maximum tilt was 4 deg and decreased with increasing reaction time, making the perceptual task more difficult the slower the response (see Decay, below). After stimuli removal, a white bar appeared that was tilted clockwise or counterclockwise by 4 deg relative to vertical. Using two different buttons on a keyboard, participants could alternate between these two response options and select the option that they thought corresponds to the tilt direction of the grating. They received one score point for a correct response. Feedback about the current score and overall score (e.g., “+1 | 7”) was provided at the end of a trial for 550 ms. If participants selected the wrong disc, no grating was displayed. A response bar appeared nonetheless, forcing participants to guess. In irrelevant trials, no response bar appeared and thus no feedback was displayed.

In relevant trials of the face block, the face of a well-known person was displayed centered on the target disc once the target disc was selected. Gray-scale images were circular with a diameter of 2.42 deg and an additional annulus of 0.14 deg in which images faded into the background of the gray target disc. We selected images from the internet with a frontal perspective and with a neutral or smiling facial expression. The 96 faces used in the experiment were randomly selected from a set of 200 images (100 males, 100 females). The depicted persons included German and international actors, musicians, show masters and politicians. The reason for using images of well-known people was the possibility to recognize a person. Image quality depended on the reaction time in the respective trial such that images degraded with increasing reaction time (see Decay, below). To this end, the images were a weighted average between the face images and a noise image in which every pixel was randomly assigned a value ranging from black to white. The relative weight given to the noise image increased with increasing reaction time. In irrelevant trials, a Gabor patch with a horizontal orientation appeared at the target disc upon selection. No noise manipulation was applied. No stimulus appeared if participants selected the wrong disc.

### Decay

In relevant trials, reward, tilt angle or image quality decayed with increasing reaction time (Fig. 1c). An exponential function yielded a decay factor *y* for any latency *t*. Decay factors were values between 0 and 1 and were multiplied with the maximal reward (0.1€), the maximal tilt angle (4 deg) or determined the relative weight of the face image (with [1 − *y*]/2 being the weight given to the noise image). The underlying decay function was the same in all three blocks and can be described as:

1 $$ y\left(t,f\right)={e}^{-\left(\frac{t-{t}_{min}}{f\ast {t}_{min}}\right)}. $$

For *t* > *t*_*min*_, *y* values were below one. For *t* < *t*_*min*_, *y* was set to 1. The *t*_*min*_ parameter was fixed at 100 ms. Thus, reward, tilt angle and image quality started to decay for latencies above 100 ms. The strength of the decay depended on the *f* parameter, which was determined by the mean latency in the last eight trials. This assured a comparable difficulty throughout the experiment and across participants. At block beginning, *f* was set to 1.88, corresponding to a mean latency of 230 ms. Mean latencies and *f* values were linearly related (Fig. 1c). For example, the *f* parameter was set to 0.44 (3.32) for a latency of 130 ms (330 ms). Participants were informed about the decay before the experiment.

### Data analysis

Saccade onsets were defined using the EyeLink 1000 algorithm. We compared mean latencies of correct trials using a 3 × 2 repeated-measures analysis of variance (ANOVA), with the factors block (reward, task, face) and relevance (relevant, irrelevant). Latency differences between relevant and irrelevant trials within a block were compared using nonparametric Wilcoxon tests.

To analyze accuracy time courses, we used the SMART procedure (smoothing method for the analysis of response time courses; van Leeuwen et al., 2019), where (i) the individual data is temporally smoothed, (ii) a weighted time course is constructed that considers the distribution of individual data, and (iii) a cluster-based permutation test is performed to compare time courses. Data were analyzed at a 1-ms resolution. We smoothed the data with a Gaussian kernel of 16-ms width and used 1,000 permutations for every test. Time courses were compared in a time window between 0 and 350 ms after target onset (unless noted otherwise). We report four values for every comparison: the *p* value, the cluster strength of the observed data (*t*), the time window of the (strongest) cluster in the observed data, and the 95^th^ percentile of the distribution of cluster strengths that result from permutation. The latter is the critical *t* value (*t*_*crit*_) to which the cluster strength of the observed data is compared. The *p* value is given by the relative position (i.e., percentile) of the observed cluster strength in the distribution of all permuted cluster strengths.

Cluster-permutation tests thus replace multiple comparisons (e.g., one *t* test for every time point) with a single comparison: the test statistic of the (strongest) observed cluster relative to the cluster strengths obtained from random permutations, where a cluster is defined as all adjacent time points that show a significant effect (*p* < .05). Hence, the cluster strength (*t*) is given by the sum of all *t* values within the cluster. This cluster test statistic is compared with a distribution of cluster strengths that was obtained from permutation: first, the labels assigning trials to conditions are randomly perturbed. Second, the strongest cluster is determined for the perturbed data. Third, steps one and two are repeated multiple times. If the cluster strength of the observed data is larger than 95% of the clusters obtained from permutation (*t > t*_*crit*_), then two time courses are assumed to differ. Please note that this analysis allows to infer that two time courses differ but not when they do so (Maris & Oostenveld, 2007; Sassenhagen & Draschkow, 2019).

We measured saccade deviation away from the distractor as an index of distractor suppression. If the target appeared, for example, at the upper right disc, a distractor at the upper left disc would be considered a counterclockwise distractor, whereas a distractor at the lower right disc would be considered a clockwise distractor. For salient distractors that appear at the same time as the target, early responses deviate towards the distractor. Thus, end points are biased towards the distractor and saccade curve towards it. The opposite can be observed long-latency saccades. Typically, the transition from deviation towards to deviation away can be observed with latencies of around 200 ms (McSorley et al., 2006; Wolf & Lappe, 2020). Yet in our paradigm, the distractor preceded the target by 187 ms, and deviation away can be observed even for early reaction times. We therefore analyzed saccades with a reaction time between 80 and 400 ms. Furthermore, for this analysis we only considered correct trials where (i) gaze was less than 2 deg away from the fixation cross at saccade onset, (ii) less than 4 deg away from target center at saccade offset (disc radius was 2 deg) and (iii) where the gaze shift from fixation cross to target was achieved by a single saccade. Saccades with missing data (due to blinks) were discarded. In total, 69.1% of trials were considered for the analysis.

Saccade trajectories were first coded relative to the gaze position at saccade beginning. In a second step, trajectories were rotated to correspond to a rightward saccade. Specifically, trajectories were rotated by 315 deg if the target was at the upper right disc, 225 deg for targets at the upper left, 135 deg for the lower left and 45 deg for targets at the lower right disc. Consequently, the vertical position of a rotated saccade corresponds to the saccade’s deviation. In a third step, we normalized the saccade duration to have the same amount of data points for each saccade. To this end, we sampled each trajectory at 25 time points using linear interpolation. In a fourth step, we computed the area under the saccade trajectory as an index of deviation (Fig. 7b). This index reflects deviation due to differences in curvature and/or end points. For a counterclockwise distractor, the deviation index was recoded (multiplied with −1) so that positive deviation indices always reflect deviation away from the distractor. Deviation indices were compared using a 3 × 2 repeated-measures ANOVA, with the factors block (reward, task, face) and relevance (relevant, irrelevant). The direction of main effects was compared using Bonferroni corrected post hoc *t* tests. One participant was discarded from the ANOVA, because this participant had less than five trials available in one of the six conditions. Including/removing this participant did not affect any conclusion drawn from the data. We additionally compared deviation index time courses using the SMART procedure with a Gaussian kernel of 32-ms width.

### Drift diffusion modelling

The drift diffusion model was fit using fast-dm-30 (Voss & Voss, 2007; Voss et al., 2015). We coded the data such that the upper threshold was associated with the correct response and the lower baseline with any error. The model comprised the four main parameters boundary separation, starting point, drift rate, and nondecision time (*a*, *z*, *v*, *t0*) as well as variability parameters of the latter three (*sz*, *sv*, *st0*). Drift rate, boundary separation and nondecision time were allowed to vary across the six conditions. Although the selective influence of latent variables on a single model parameter has been challenged (e.g.,Dutilh et al., 2019 ; Rae et al., 2014), emphasizing speed or accuracy are most consistent with changes in the boundary separation parameter (Ratcliff & Rouder, 1998; Voss et al., 2004). However, we additionally analyzed nondecision times as a function of the experimental conditions because accuracy instructions have been shown to also affect nondecision time parameters (Dutilh et al., 2019; Rae et al., 2014). The starting point is typically affected by prior information (e.g., Dutilh et al., 2019; Mulder et al., 2012) and indicative of a response bias. A bias may occur if the target appeared more frequently at one location, or if the reward had been larger for a particular location. For reasons of parsimony, we therefore decided to not vary the starting point parameter across conditions, because the location of stimuli was fully balanced, and participants had no prior information that would bias their responses towards any location.

We used the Kolmogorov–Smirnov statistic for parameter optimization (Voss & Voss, 2007). The drift rate is normally distributed with mean *v* and standard deviation *sv*, whereas the variability of starting point and nondecision time follow a uniform distribution with means *z* and *t0* and width *sz* and *st0* (Voss et al., 2015). Boundary separation, nondecision time and drift rate parameters were compared using a 3 × 2 repeated-measures ANOVA, with the factors block (reward, task, face) and relevance (relevant, irrelevant).

### Experiment 2: Target facilitation versus distractor suppression

To distinguish whether motivation by reward affects target facilitation or distractor suppression, we conducted Experiment 2, where the distractor was absent in half of the trials. We recorded data of 36 participants (age range: 18–29 years, 30 females). The experiment consisted of one block of 640 trials. Each block contained the same number of trials with and without distractor as well as the same number of trials with and without reward (2 × 2 design). The trial procedure for trials with a distractor was equivalent with the trial procedure of the reward block of Experiment 1: A text at the beginning of each trial indicated whether the next trial rewarded (“reward”) or unrewarded (“no reward”). Four blue discs appeared. After a uniform random time interval between 500 and 1,000 ms one of the discs turned white. This was the distractor. After additional 187 ms, the target disc turned gray. Unlike Experiment 1, distractor and target were spatially independent. Thus, the target could appear opposite to the distractor, at one of the two neighboring locations or at the same location, in which case it replaced the distractor. Importantly, for the analysis we only considered trials in which distractor and target location did not coincide. In trials without distractor, the target disc turned gray after the uniform random interval between 500 and 1,000 ms. The different trial types were randomly interleaved.

Saccade latencies were compared using a 2 × 2 repeated-measures ANOVA with the factors distractor presence (present vs. absent) and reward (reward vs. no reward). The analysis of accuracy time courses was equivalent to the main experiment, except that the analysis was restricted to a time window between 80 to 300 ms after target onset in the distractor absent condition, and between 0 and 300 ms after target onset in the distractor present condition. We computed the deviation index for every saccade, consistent with Experiment 1. Thus, for this analysis we only considered distractor-present trials where the distractor was at a neighboring position (clockwise or counterclockwise). Deviation indices in rewarded and unrewarded trials were compared using a paired *t* test. One participant was not considered for this comparison because of less than five trials in one condition. Time courses of deviation indices were compared using the SMART procedure in a time window between 80 and 300 ms using a Gaussian kernel with a width of 32 ms.

## Results

### Speed

**Fig. 2:**
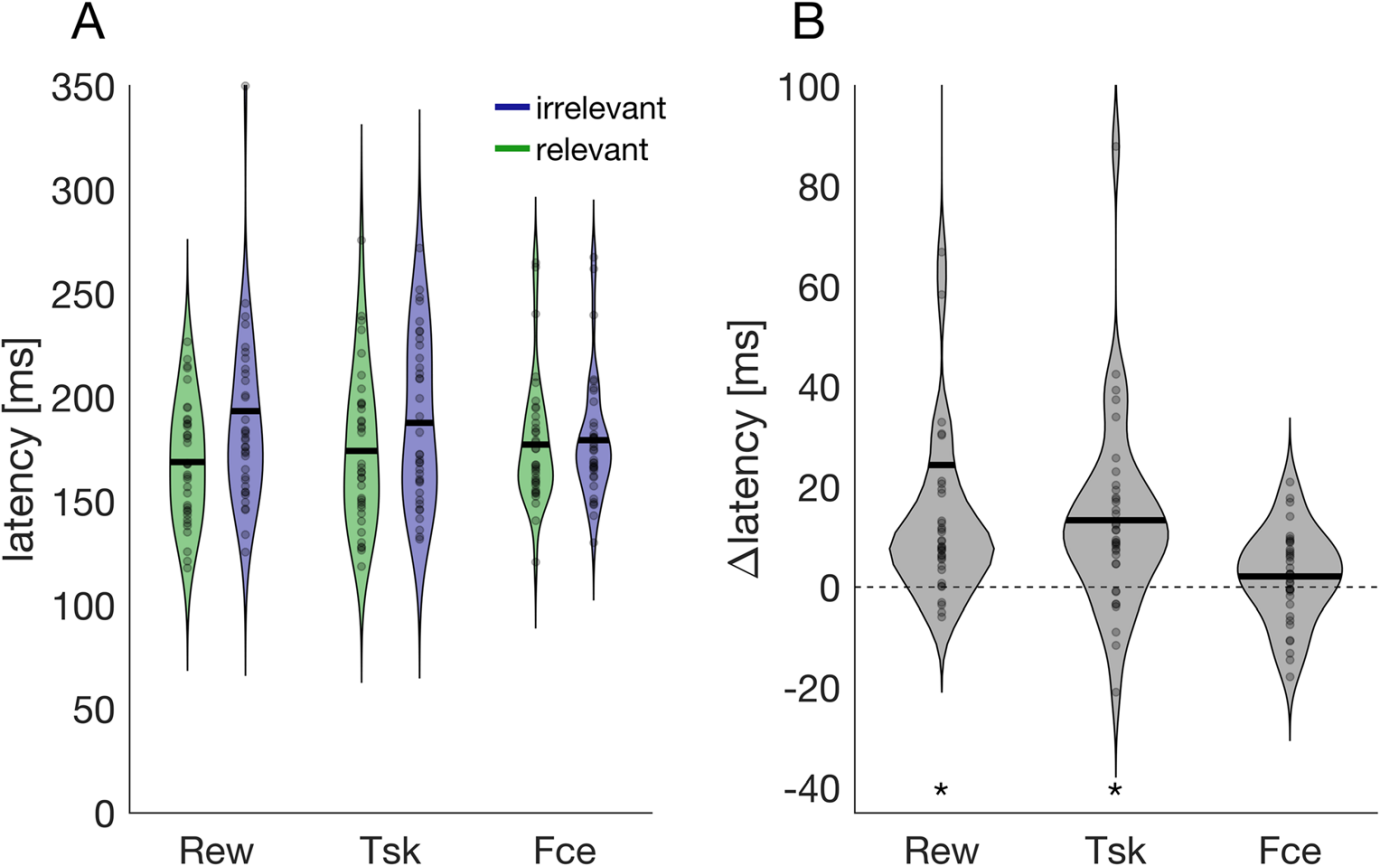
Speed. **a** Violin plots of individual latencies for the three different blocks (Rew = Reward, Tsk = Task, Fce = Face). Data points (gray dots) in the left panel denote the mean of an individual, horizontal black lines denote the mean across all participants. Green colors are relevant trials (reward, task, seeing a face) whereas blueish colors are irrelevant trials (no reward, no task, seeing a grating). To enhance visibility, one value in the irrelevant/reward condition (413 ms) is outside the plotted range. **b** Latency difference between irrelevant and relevant trials in (**a**) with positive values denoting higher latencies in irrelevant trials. Asterisks indicate a value significantly different from 0. Two values in the reward condition are outside the plotted range (168 ms, 278 ms). (Color figure online)

To analyze how the three different consequences affect the speed and accuracy of target selection, we analyzed saccade latencies of correct responses as an index of speed, and the proportion of trials in which the correct disc was selected as an index of accuracy. Figure 2 shows latencies and their variability in the three different blocks and the two levels of relevance, respectively. Descriptively, mean latencies in relevant trials were lowest in the reward block, *M*_*rew*_ = 169.0 ms, intermediate in the task block, *M*_*tsk*_ = 174.3 ms, and highest in the face condition, *M*_*fce*_ = 177.4 ms. The opposite pattern was observed in irrelevant trials (*M*_*rew*_ = 193.4 ms, *M*_*tsk*_ = 187.7 ms, *M*_*fce*_ = 179.5 ms). This was also reflected in the Block × Relevance interaction, *F*(2, 70) = 4.63, *p* = .013, ${\upeta}_p^2$ = 0.117. Comparing relevant and irrelevant trials within each block, yielded significantly lower latencies in relevant trials for the reward block, *Z* = 4.78, *p* < .001, and the task block, *Z* = 4.02, *p* < .001, but not for the face block, *Z* = 1.48, *p* = .140. The ANOVA also revealed a main effect of relevance, *F*(1, 35) = 14.55, *p* < .001, ${\upeta}_p^2$ = 0.294, highlighting the lower latencies in relevant trials, but no main effect of block, *F*(2, 70) = 0.088, *p* = .8916, ${\upeta}_p^2$ = 0.003. Thus, we found faster responses when obtaining monetary rewards or task-relevant information. However, seeing a face versus seeing an irrelevant grating did not affect saccade latencies.

### Accuracy

**Fig. 3:**
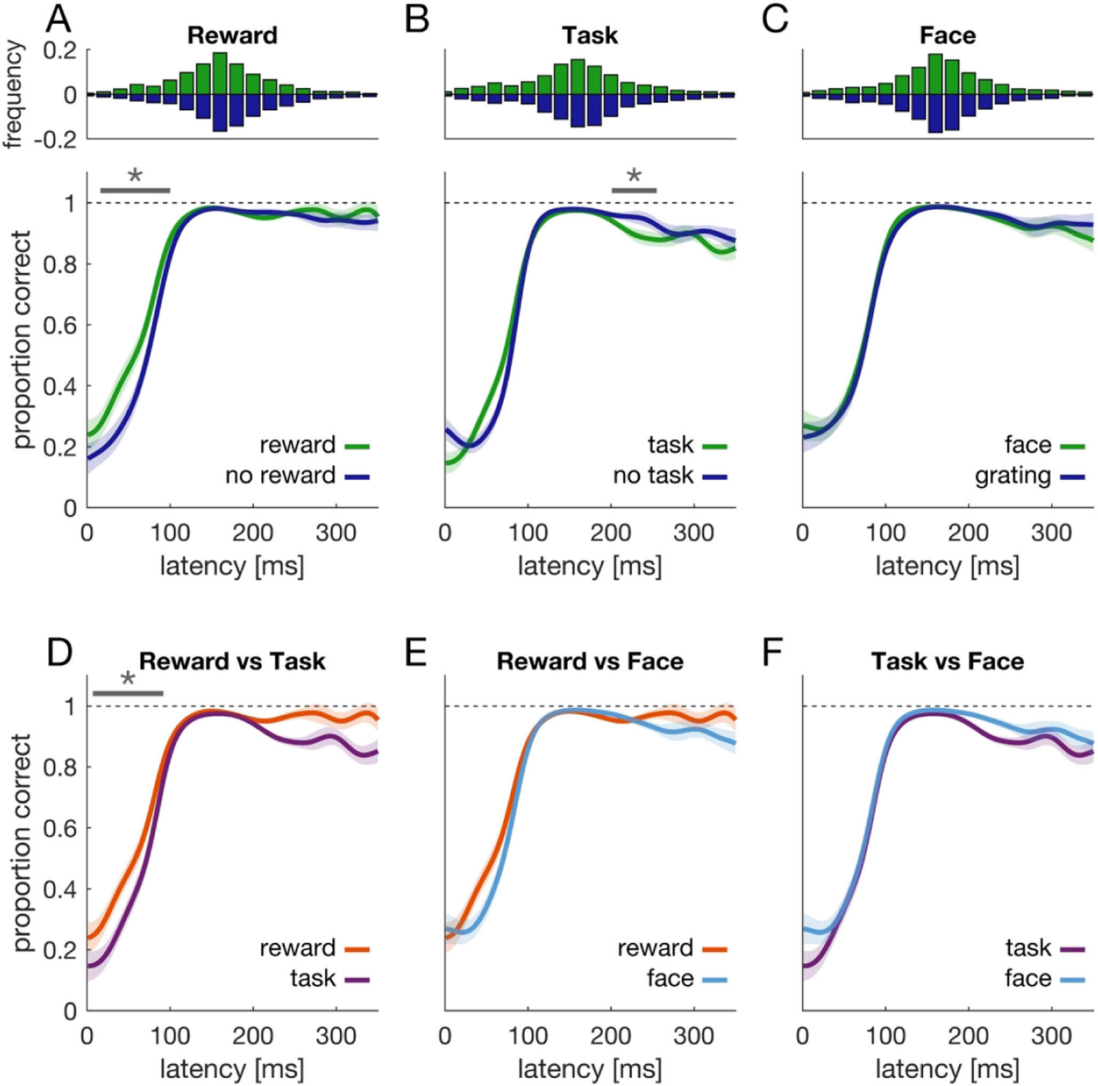
Accuracy. **a**–**c** The lower panels show accuracy time courses for relevant (green) and irrelevant (blue) trials in the three blocks, respectively. Each time course shows the proportion of saccades to the correct disc, the target, as a function of saccade latency. Shaded regions denote the 95% confidence interval that results from comparing the two time courses against each other (van Leeuwen et al., 2019). Gray horizontal lines and asterisk indicate a significant cluster. Upper panels show reaction time histograms pooled across all participants. **d**–**f** Accuracy time courses comparing the relevant conditions (i.e., green data) from **a**–**c**. (Color figure online)

The bottom panels in Fig. 3a–c show accuracy time courses for relevant and irrelevant trials in the three blocks, respectively. Common across all conditions is that response accuracy increases with reaction time and reaches an asymptote approximately 120 ms after target onset. We compared these time courses of proportion correct responses for relevant versus irrelevant trials in the respective blocks (Fig. 3a–c). Time courses in the reward block differed, *t* = 250.7, *t*_*crit*_ = 149.3, *p* = .002. The cluster was found in a time window 16–100 ms after target onset (Fig. 3a). During this time window, performance was superior for relevant compared with irrelevant trials.

The opposite was found in the task block (Fig. 3b). One significant cluster was detected (201–255 ms) in which performance was superior for irrelevant compared with relevant trials, *t* = 147.2, *t*_*crit*_ = 142.8, *p* = .042. Furthermore, comparing relevant trials in the reward block with those from the task block (green line in Fig. 3a versus green line in Fig. 3b), revealed better performance in the reward block, *t* = 251.2, *t*_*crit*_ = 149.4, *p* = .002, time window: 7–92 ms (Fig. 3d). No such difference was observed when comparing relevant and irrelevant trials from the face block (Fig. 3c) or when comparing relevant trials from the face block with relevant trials from either of the other blocks (Fig. 3e–f; *p* > .3).

Aggregated across time, we observed no significant performance benefit for relevant compared with irrelevant trials in the reward block (0.91 vs. 0.90), *Z* = 0.24, *p* = .813. Although we observed an accuracy benefit for early responses, it does not show on the aggregated level, possibly because of the higher number of short-latency responses in relevant compared with irrelevant trials (22% vs. 17% of trials with latencies <120 ms). Thus, due to the reaction time difference, a higher number of responses in relevant trials were carried out in a time window where an incorrect response was more likely. In the task block, we observed a lower response accuracy for relevant compared with irrelevant trials (0.84 vs. 0.87), *Z* = −2.26, *p* = .024, whereas no difference between relevant and irrelevant trials was observed in the face block (0.90 vs. 0.89), *Z* = 0.62, *p* = .536.

In sum, our results are most consistent with (i) faster performance when expecting a monetary reward and an accuracy benefit for early responses, (ii) faster but less accurate performance with a perceptual task, and (iii) no difference in either speed or accuracy when seeing a human face or an otherwise irrelevant grating.

### Error analysis

**Fig. 4:**
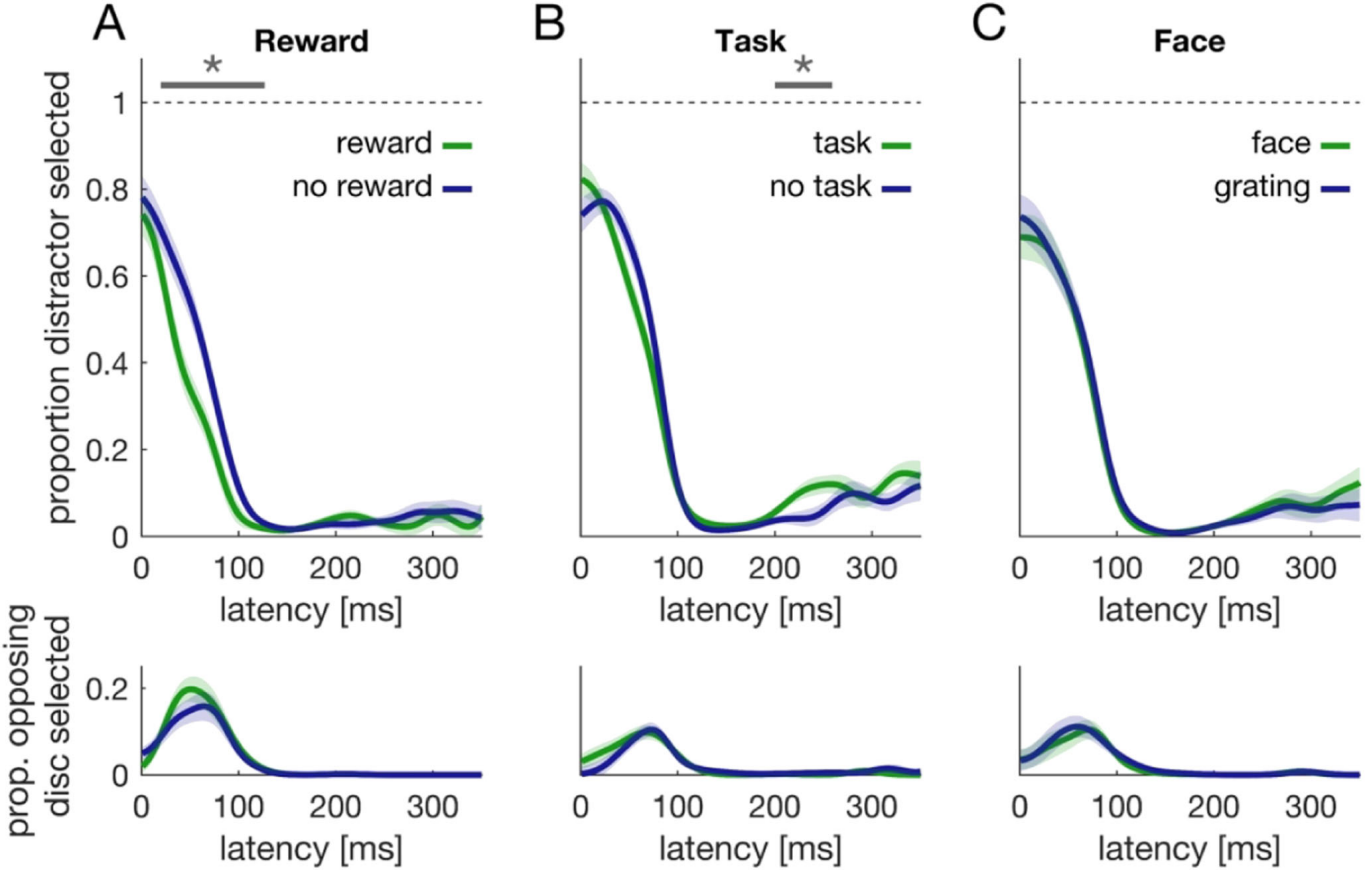
Error analysis. Proportion of erroneous responses over time for relevant (green) and irrelevant (blue) trials in the three blocks. The upper row shows trials in which the distractor was selected, whereas the lower row shows trials in which the disc opposing the target was selected. Given that the onset of the distractor renders two discs possible target locations (the target disc and the opposing disc), the latter can be seen as an index of strategic anticipation. Asterisks and solid gray lines indicate clusters, and shaded regions denote the 95% confidence interval that results from comparing relevant and irrelevant time courses (van Leeuwen et al., 2019). (Color figure online)

**Fig. 5:**
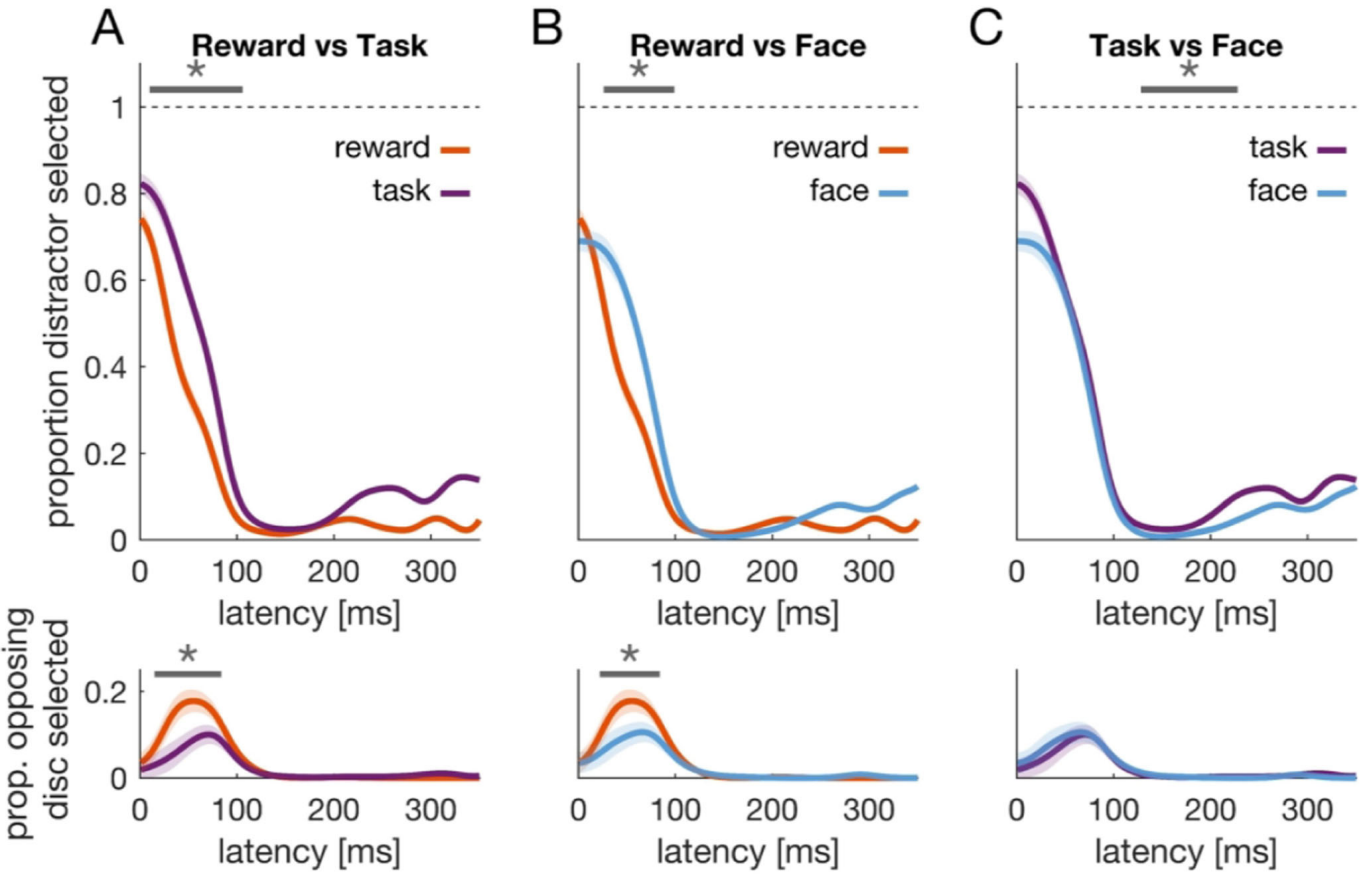
Error analysis across blocks. Top row: Time courses for erroneous responses to the distractor comparing the relevant conditions (i.e., green data) from the top panels of Fig. 4. Bottom row: Time courses for erroneous responses to the opposing disc comparing merged data from relevant and irrelevant trials (i.e., green and blue data) from bottom panels in Fig. 4. (Color figure online)

In a next step, we wanted to know what determines the differences in accuracy. To this end, we analyzed the time course of errors (Figs. 4 and 5). Particularly, we looked at two different kinds of errors. First, responses to the distractor disc. These errors would be indicative of a premature response triggered by the strong luminance transient (Wolf & Lappe, 2020; Yantis & Jonides, 1984). Second, we analyzed saccadic responses to the opposing disc. Given that distractor and target disc were always next to each other, knowing the location of the distractor renders two discs possible target locations. We refer to this second disc who did not turn into the target as opposing disc (righthand panel in Fig. 1a). These errors would reflect target anticipation and thus be possibly indicative of strategic behavior. For example, an anticipatory saccade to either of the two potential target discs around the time of target onset (or even before) might yield the maximal reward—yet only with a chance of 50%. Hence, errors might be due to this strategic gambling behavior.

Figure 4 shows error time courses for responses to the distractor disc (upper row) and responses to the opposing disc (lower row). The proportion of trials in which the distractor was selected as saccade target is high for early responses and then decreases with increasing latency. In the reward block we found a significant cluster in a time window 20–127 ms after target onset (Fig. 4a, upper panel), *t* = 368.7, *t*_*crit*_ = 152.6, *p* < .001. During this cluster, there were fewer error responses for relevant compared with irrelevant trials. This is consistent with the pattern observed in accurate trials (Fig. 3). In the task block, error time courses were also consistent with the accuracy data. Relevant and irrelevant trials differed, *t* = 184.0, *t*_*crit*_ = 136.3, *p* = .018. The corresponding cluster was detected 200 – 259 ms after target onset. In this time window, we observed more errors in trials with a perceptual task (relevant) than without (irrelevant). In the face block, no cluster (and thus no difference) between relevant and irrelevant trials was detected. Hence, the test-statistic *t* is effectively zero (*t* = 0) and smaller than the critical value of *t*_*crit*_ = 148.7.

When comparing relevant trials of different blocks in term of erroneous responses to the distractor (Fig. 5, upper row), we found that data from the reward block differed from the other two, task: *t* = 423.5, *t*_*crit*_ = 166.2, *p* < .001, 10–106 ms, face: *t* = 253.6, *t*_*crit*_ = 173.0, *p* = .006, 26–99 ms. In each case, fewer errors were found in the reward block during the detected clusters. Moreover, time courses of relevant trials differed in the task versus the face block, *t* = 238.0, *t*_*crit*_ = 141.3, *p* = .004, 128–228 ms, with more errors in the task block during the detected cluster.

For error responses to the opposing disc, no clusters were detected when comparing relevant and irrelevant trials in each of the three blocks. This analysis was restricted to the first 150 ms after target onset, because hardly any of these errors occurred after this time point. However, we observed a difference between blocks (Fig. 5, lower row). The reward block differed from the task block, *t* = 224.7, *t*_*crit*_ = 123.5, *p* < .001, 15–84 ms, as well as from the face block, *t* = 171.3, *t*_*crit*_ = 109.3, *p* = .009, 22–84 ms. In both cases, erroneous responses to the opposing disc were more pronounced in the reward block.

To summarize, we found more errors due to strategic anticipation in the reward block than in the other two blocks (Fig. 5, lower row). Yet, relevant trials of the reward block were characterized by fewer erroneous responses to the distractor (Figs. 4 and 5, upper row). The difference in response accuracy between relevant and irrelevant trials (Fig. 3) was better explained by avoiding a response towards the distractor than by differences in target anticipation.

### Drift diffusion modelling

**Fig. 6:**
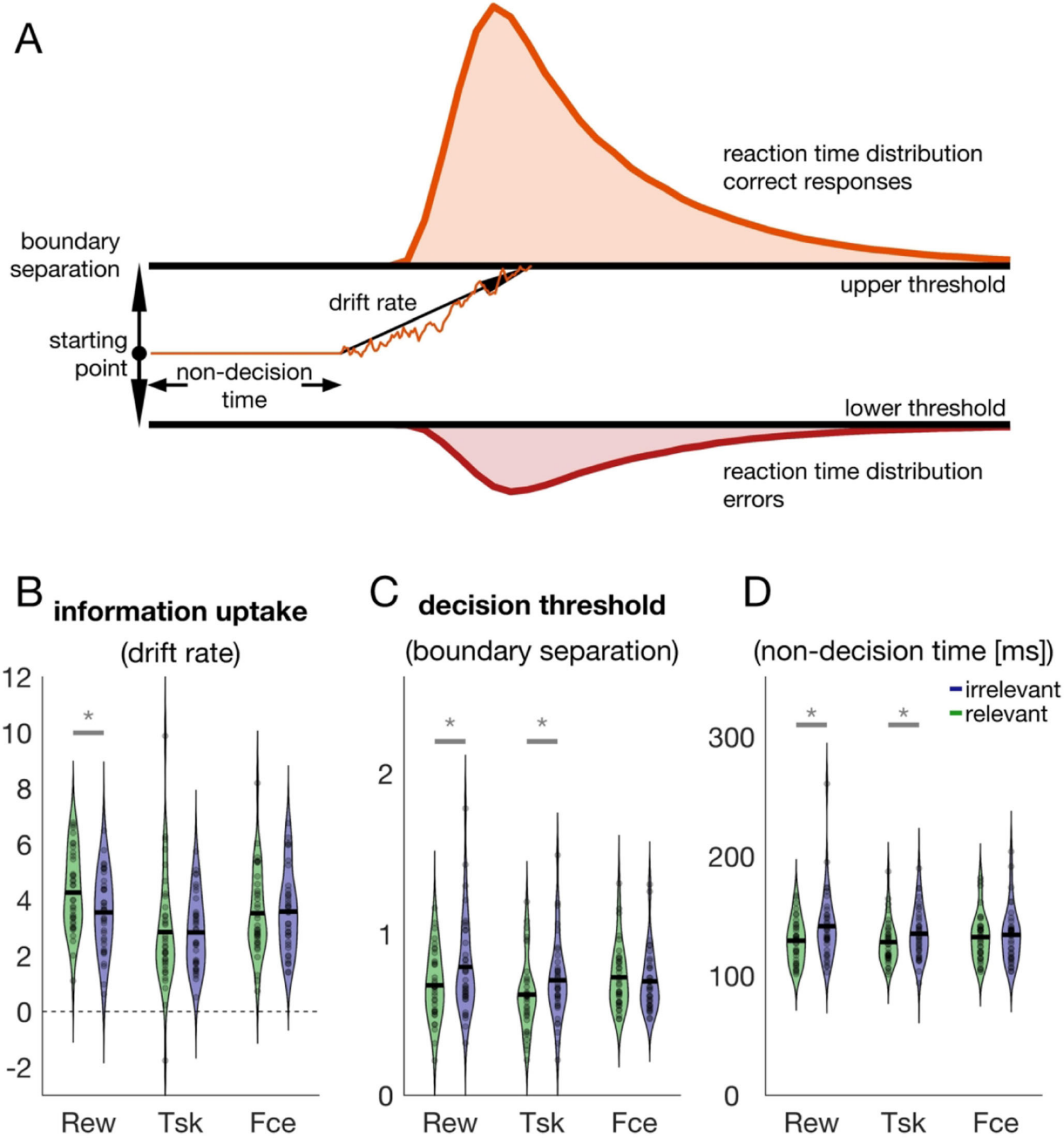
Drift diffusion model. **a** Illustration of the drift diffusion model. The model assumes that a response is made once the accumulation process reaches either of two boundaries. Each boundary is associated with a different response (here: correct response versus error). The systematic component of the drift process is the drift rate (i.e., mean evidence uptake per time), whereas the random component is reflected by noise. The thin orange line denotes an example trial, and the colored areas denote latency distributions for correct trials (orange) and errors (red). **b**, **c**, **d** Violin plots of drift rate (**b**), boundary separation (**c**), and nondecision time parameter (**d**). Gray lines and asterisks indicate a significant different between relevant and irrelevant trials of a particular block. (Color figure online)

Speed–accuracy trade-offs can be captured by sequential sampling models. We therefore complemented our analysis with a drift diffusion modelling approach to reveal how the different consequences affect latent decision variables. The drift diffusion model (Ratcliff, 1978; Ratcliff & Tuerlinckx, 2002; Fig. 6) allows to infer these latent decision variables based on the joint modelling of reaction times on the one hand and either free binary decisions or correct/incorrect responses on the other hand. The model assumes that evidence starts to accumulate in between two boundaries until one of the boundaries is reached. The systematic component of the evidence accumulation process is called the drift rate. It denotes the mean evidence uptake per time. Yet evidence accumulation is noisy. Therefore, even if the drift rate favors one of the two decision outcomes, the other threshold can be reached first due to the noise. The drift rate can be affected by changing the difficulty of the task—for example, by decreasing or increasing the target’s signal to noise ratio. Therefore, the information uptake is sometimes referred to as the ease of processing. Decision threshold on the other hand is reflected in the boundary separation parameter. This parameter can be affected by instructing the participant to either emphasize speed or accuracy. Thus, the boundary separation parameter captures trade-offs in speed and accuracy. Increasing the boundary separation would reduce the number of errors but would also increase reaction times. The other two main parameters are the starting point and the nondecision time. The former can capture response biases that can occur if one of the response options is more likely or associated with a higher payoff (e.g., Dunovan et al., 2014; Mulder et al., 2012). In our paradigm this would have been the case if the target was not equally distributed across the four discs or if one of the discs was associated with a higher reward than the other discs. The nondecision time parameter is thought to capture all aspects of the reaction time that is not devoted to the decision itself but devoted to other processes, like sensory encoding and motor execution. However, the nondecision time has been reported to be also affected when accuracy or speed is emphasized (Dutilh et al., 2019).

We fit the full drift diffusion model to the data and allowed drift rate, boundary separation and nondecision time to vary across condition. To assess whether our manipulation affected decision thresholds, we compared boundary separation parameters using a 3 × 2 repeated-measures ANOVA (Fig. 6c). The ANOVA revealed a main effect of relevance, *F*(1, 35) = 5.70, *p* = .022, ${\upeta}_p^2$ = 0.140, as well as a block × relevance interaction, *F*(2, 70) = 4.44, *p* = .015, ${\upeta}_p^2$ = 0.113: Boundary separation was lower with a reward compared with no reward, *t*(35) = 2.36, *p* = .024, *d* = 0.394, and it was lower with a task compared with no task, *t*(35) = 2.69, *p* = .011, *d* = 0.448 We found no difference in boundary separation when the consequence of a successful saccade was either a face or a grating, *t*(35) = 0.83, *p* = .412, *d* = 0.138.

We additionally analyzed nondecision time parameters because emphasizing accuracy has been shown to also affect nondecision times in addition to the boundary separation (Dutilh et al., 2019). The observed pattern in nondecision times (Fig. 6d) was mostly consistent with the pattern of results observed in the boundary separation parameter: There was a main effect of relevance, *F*(1, 35) = 17.54, *p* < .001, ${\upeta}_p^2$ = 0.334, but no Block × Relevance interaction, *F*(2, 70) = 2.97, *p* = .058, ${\upeta}_p^2$ = 0.078. Nondecision times were lower with a reward compared with no reward, *t*(35) = 2.70, *p* = .005, *d* = 0.45, and with a task compared with no task, *t*(35) = 3.59, *p* < .001, *d* = 0.598. Again, we found no difference between the two conditions of the face block (face vs. grating), *t*(35) = 1.32, *p* = .098, *d* = 0.219. This suggests that relevant and irrelevant trials differed in decision thresholds and thus that participants behaved less cautious when they expected a monetary reward or a perceptual task.

We next analyzed drift rate parameters to reveal whether conditions differ in information uptake (Fig. 6b). Typically, drift rates are expected to be higher when the task easy, for example because the target has a higher contrast and can be more easily processed. The ANOVA on drift rates revealed a main effect of block, *F*(2, 70) = 7.53, *p* = .001, ${\upeta}_p^2$ = 0.177. Descriptively, mean drift rates were highest in the reward block, *M*_*rew*_ = 3.91, intermediate in the face block, *M*_*fce*_ = 3.56, and lowest in the task block, *M*_*tsk*_ = 2.85. Drift rates in the task block were significantly lower than drift rates in the reward block, *t*(35) = 3.81, *p* < .001, *d* = 0.635, or the face block, *t*(35) = 2.37, *p* = .023, *d* = 0.395. There was no difference between the reward and face block, *t*(35) = 1.37, *p* = .178, *d* = 0.229. Most importantly, we observed a Block × Relevance interaction, *F*(2, 70) = 3.27, *p* = .044, ${\upeta}_p^2$ = 0.085: Whereas drift rates were higher with a reward compared with no reward, *t*(35) = 2.811, *p* = .008, *d* = 0.468, there was no difference in drift rates between the task and no task condition, *t*(35) = 0.043, *p* = .966, *d* = 0.007, or between the face and grating condition, *t*(35) = 0.255, *p* = .800, *d* = 0.042.

In sum, drift diffusion modelling revealed that relevant and irrelevant trials of the reward block (reward vs. no reward) and task block (task vs. no task) differed in decision thresholds. Participants emphasized speeded responses when they expected a perceptual task or a monetary reward. This was reflected in the boundary separation parameter. The same pattern was observed in nondecision times. On the other hand, a difference in information uptake between relevant and irrelevant trials was only observed in the reward block. This was reflected in drift rates.

### Saccades deviate away from distractor locations

**Fig. 7:**
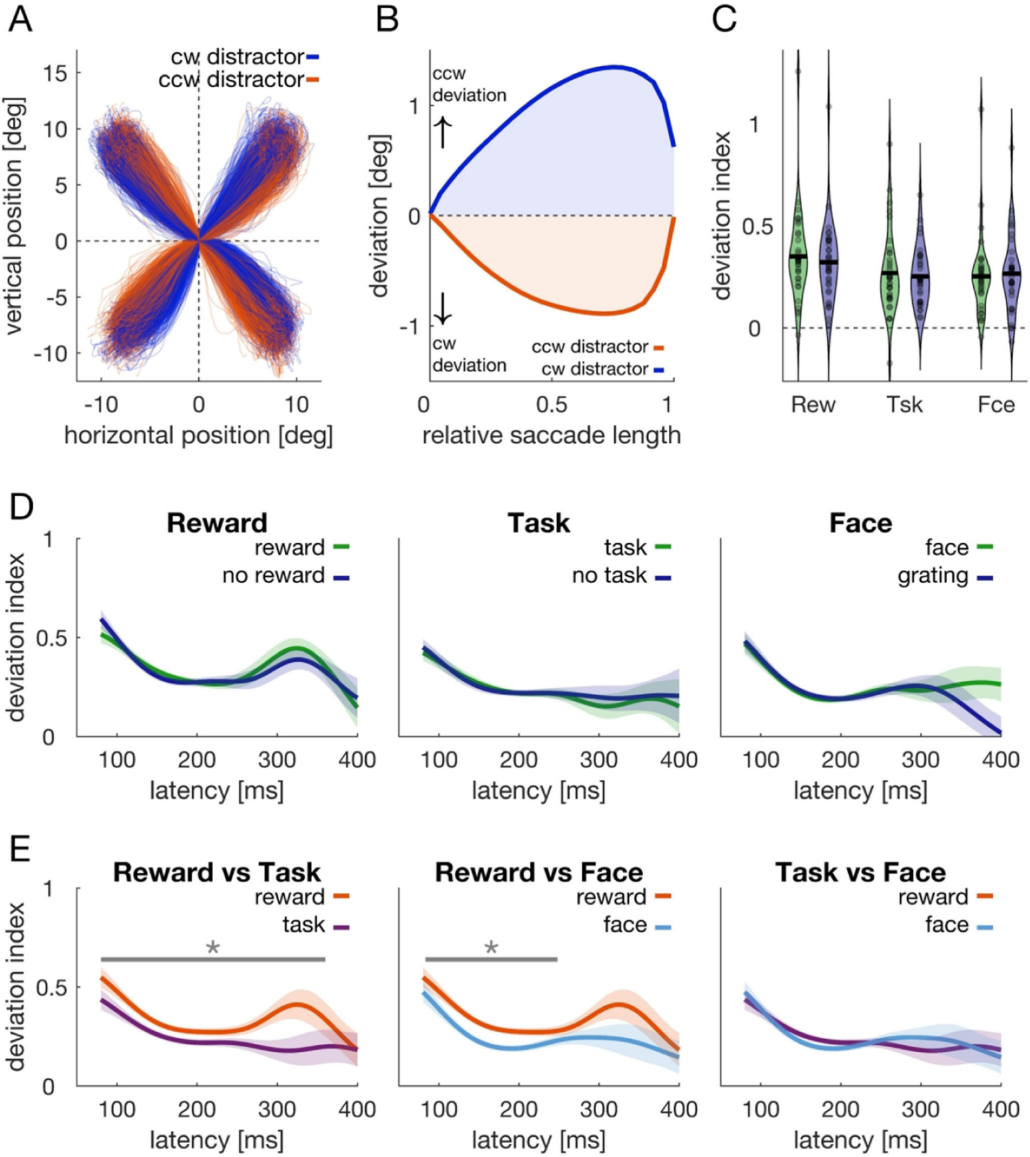
Saccades deviate away from distractor locations. **a** Saccade trajectories of all conditions to the four different target locations when the distractor was at a neighboring location, i.e., clockwise (blue data) or counterclockwise by 90° (orange data). The data was coded relative to saccade starting points. **b** Computation of deviation index. Saccades were normalized in length and rotated such that the straight connection between fixation cross and target center was purely horizontal. Consequently, any deviation in saccade trajectories can be found along the vertical dimension. For each saccade, we computed a deviation index as the area under the saccade trajectory (blue and orange shaded area). For a counterclockwise distractor, values were recoded (multiplied with -1) so that positive deviation indices denote deviation away from the distractor. **c** Violin plots of the mean deviation index in the respective conditions. Indices were different from 0 and larger in the reward block. **d** Deviation indices as a function of saccade latency (SMART analysis) comparing relevant (green) and irrelevant trials (blue) from the respective blocks. Shaded regions denote the 95% confidence interval that results from comparing the two time courses against each other (van Leeuwen et al., 2019). **e** Comparison of merged data from relevant and irrelevant trials (i.e., green and blue data from **d**). Gray lines and asterisks denote a significant cluster. (Color figure online)

To test whether the behavioral results can be explained by differences in distractor suppression, we analyzed saccade deviation as a function of distractor position. We made use of the fact that long-latency saccades curve away from distractors (McSorley et al., 2006; Mulckhuyse et al., 2009), reflecting distractor suppression (for review, see Van der Stigchel, 2010). A similar temporal dependency reflecting distractor suppression can be observed in saccade end points (Wolf & Lappe, 2020). If the distractor in our paradigm is suppressed, we would thus expect that saccades deviate away from it, because of differences in saccadic end points as well as saccadic curvature. Given that the distractor preceded the target by 187 ms and did not appear simultaneously, even short-latency saccade showed characteristics of deviation away (Fig. 7). Figure 7a shows trajectories of saccades (80–400 ms) towards each of the four target locations when the distractor was at a neighboring position, either clockwise (blue) or counterclockwise (orange) relative to the target’s position. To analyze this data, we first rotated each saccade so that the ordinate axis codes the saccade’s deviation (Fig. 7b). Please note that this measure of deviation jointly codes deviation due to saccade curvature (McSorley et al., 2006) as well as due to differences in saccade end points (Wolf & Lappe, 2020). In a second step, we normalized saccade duration to have the same number of data points for each saccade. In a third step, we computed the area under the saccade trajectory as an index of deviation (shaded areas in Fig. 7b). We coded deviation indices so that positive values always denote deviation away from the distractor.

Deviation indices (Fig. 7c) were above zero in all conditions (*p* < .001, *d* > 1.3). The ANOVA on the deviation indices revealed a main effect of block, *F*(2, 68) = 10.45, *p* < .001, ${\upeta}_p^2$ = 0.235. Deviation indices were higher in the reward block compared with both, the task block, *t*(34) = 3.64, *p* = .003 or the face block, *t*(34) = 5.24, *p* < .001. No difference was observed between the task and face block, *t*(34) = 0.07, *p* > 0.999. We neither observed a Block × Relevance interaction, *F*(2, 68) = 1.019, *p* = .366, ${\upeta}_p^2$ = 0.029, nor a main effect of relevance, *F*(1, 34) = 1.64, *p* = .209, ${\upeta}_p^2$ = 0.046. Thus, there was also no evidence for a difference in deviation indices between rewarded and unrewarded trials, *t*(34) = 1.80, *p* = .080, *d* = 0.299.

We next analyzed deviation indices as a function of saccade latency. Consistent with the ANOVA on the aggregated values, we observed no difference between relevant and irrelevant trials in any of the three blocks (Fig. 7d, no clusters detected). Yet deviation time courses from the reward block differed from the task block, *t* = 814.1, *t*_*crit*_ = 205.6, *p* < .001, 80–360 ms, as well as from the face block, *t* = 594.4.1, *t*_*crit*_ = 221.1, *p* < .001, 83–248 ms (Fig. 7e).

### Distractor suppression versus target enhancement

To reveal whether motivation by reward facilitates target processing or whether it aids distractor suppression (Pearson & Le Pelley, 2021), we conducted Experiment 2, where the distractor was only present in half of the trials and where distractor and target were spatially independent (see Methods). If motivation by reward increases speed and accuracy by improving distractor suppression, then we would expect that performance only differs when the distractor is present. If, however, we observe a reward benefit in trials with and without distractor, this would be evidence that motivation by reward improves performance by improving target facilitation.

**Fig. 8:**
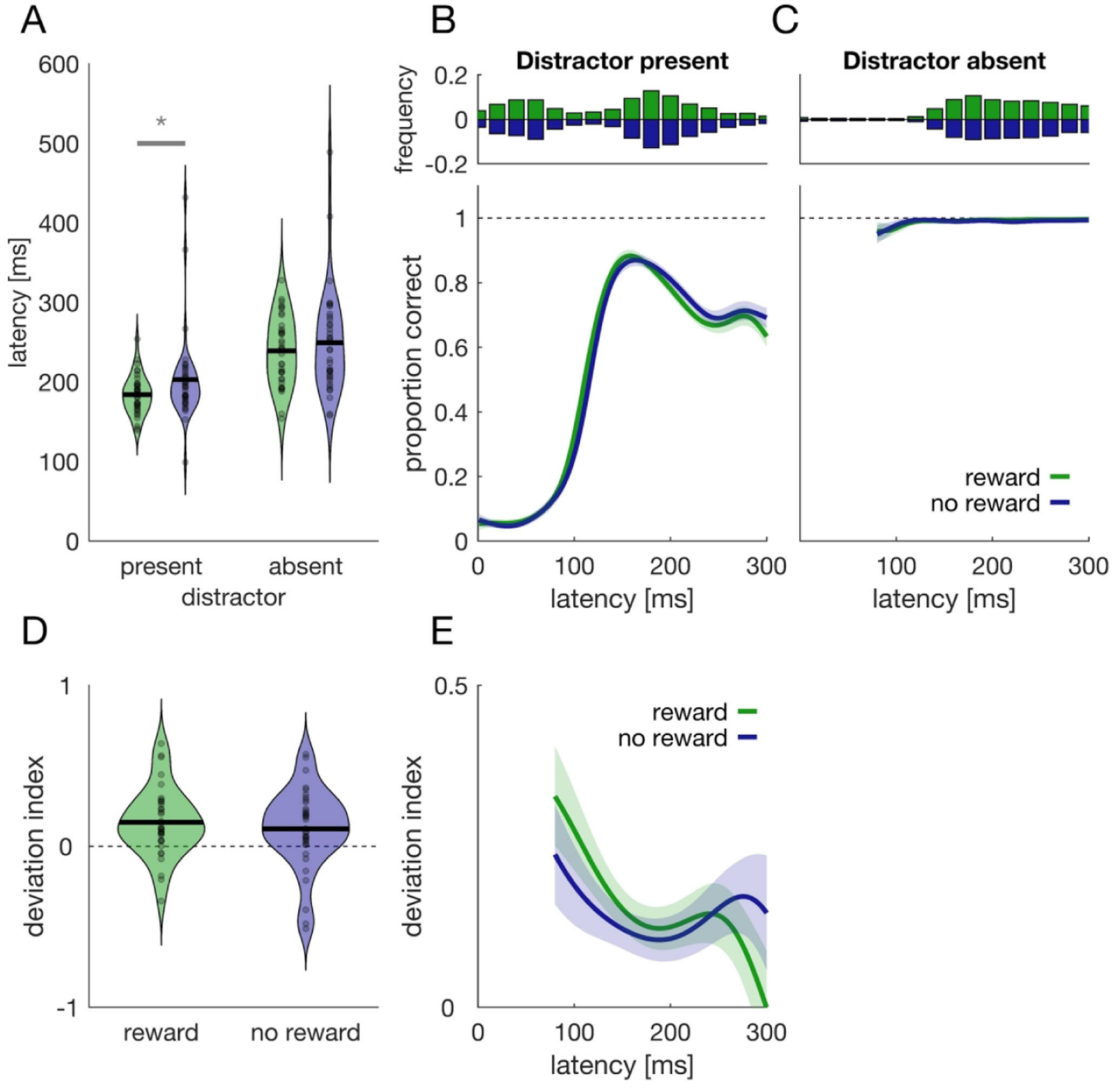
Experiment 2: Target facilitation vs. distractor suppression. **a** Violin plot of saccade latencies when the distractor was present or absent, both for the condition with reward (green) and without (blue). Gray dots represent individual values whereas black lines indicate the aggregated mean. The asterisk and horizontal gray line denote a significant comparison. **b**, **c** Time course analysis for distractor present (**b**) and absent trials (**c**). Each time course shows the proportion of saccades to the correct disc as a function of saccade latency. Shaded regions denote the 95% confidence interval that results from comparing the two time courses against each other (van Leeuwen et al., 2019). Upper panels show reaction time histograms pooled across all participants. **d** Violin plot of the deviation index in rewarded (green) and unrewarded trials (blue). **e** Deviation index as a function of saccade latency (SMART analysis). (Color figure online)

Figure 8a shows saccade latencies and accuracy time courses for distractor present versus absent trials. The ANOVA on the saccade latencies revealed a main effect of distractor presence: saccades were initiated later when the distractor was absent, *F*(1, 35) = 52.66, *p* < .001, ${\upeta}_p^2$ = 0.601. Importantly, we observed a distractor presence × reward interaction, *F*(1, 35) = 4.776, *p* = .036, ${\upeta}_p^2$ = 0.120. Latencies in the reward and no reward condition differed when the distractor was present, *Z* = 4.29, *p* < .001, but not when it was absent, *Z* = 1.49, *p* = .135. The latency results are thus more consistent with the idea that reward improves distractor suppression.

Accuracy time courses can be found in Fig. 8b–c. Whereas performance was at ceiling in distractor-absent trials, time courses increased starting from around 80 ms after target onset when the distractor was present. No cluster (and thus no difference between time courses) was detected when the distractor was absent. In distractor-present trials, we detected a cluster in a time window between 94 and 120 ms after target onset, yet we found no evidence for a difference between the time courses, *t* = 67.2, *t*_crit_ = 139.1, *p* = .289.

Consistent with Experiment 1, we computed deviation indices when the distractor was at a neighboring position. Thus, this analysis can only be conducted for distractor-present trials. Deviation indices (Fig. 8d) were positive and different from 0, both in trials with a reward, *t*(34) = 4.20, *p* < .001, *d* = 0.709, as well as in unrewarded trials, *t*(34) = 2.58, *p* = .014, *d* = 0.436. We observed no difference in deviation indices between rewarded and unrewarded trials, *t*(34) = 0.83, *p* = .413, *d* = 0.14. This was also true when deviation indices were analyzed as a function of saccade latency (Fig. 8e). No cluster was detected, and we thus observed no difference between rewarded and unrewarded trials.

## Discussion

The present results show that only motivation by reward can simultaneously increase response speed and accuracy and is thus capable of decreasing internal noise (Manohar et al., 2015). Obtaining task-relevant information increased speed but decreased accuracy, a pattern consistent with the traditional speed–accuracy trade-off. If successful saccade target selection resulted in seeing a face on the other hand, neither speed nor accuracy was affected, suggesting that motivational aspects do not contribute to earlier and/or more accurate saccades to faces, which highlights the importance of low-level information in the oculomotor selection of faces (Crouzet & Thorpe, 2011; Honey et al., 2008).

To distinguish whether motivation by reward facilitated target processing or aided with distractor suppression (Wöstmann et al., 2022), we analyzed saccadic deviation as an index of suppression (Fig. 7). Moreover, we conducted Experiment 2, where we randomly interleaved trials with and without distractor and kept distractor and target spatially independent. We again analyzed accuracy, latencies, and saccadic deviation. No difference in accuracy time courses between rewarded and unrewarded was observed, neither when the target was absent, nor when it was present. In distractor-absent trials, this can be attributed to a ceiling effect. In distractor present trials this might be explained by the more difficult task (compared with Experiment 1) and the decreased consistency between participants. Hence, accuracy data neither favored target facilitation nor distractor suppression. For saccade latencies, we found that a benefit in speed could only be observed when the distractor was present (Fig. 8a). This is more consistent with the notion that motivation by reward aided distractor suppression. The observation that latencies were shorter when the distractor was present compared with when it was absent is at odds with various findings comparing distractor present and absent trials (for review, see Gaspelin & Luck, 2018). This can most likely be explained the fact that the distractor preceded the target in our experiment. Hence, in distractor-absent trials there was uncertainty whether participants should respond to the onset of a peripheral target, because they would first have to discriminate whether this is a distractor or a target. There was no such uncertainty in distractor present trials: If the distractor was already present, then participants knew that they could respond the upcoming stimulus.

Saccade trajectories in both experiments deviated away from distractor locations (Figs. 7 and 8), suggesting that the distractor was suppressed. Deviation was stronger in the reward block compared with the other two blocks (Fig. 7), yet we observed no difference between rewarded and unrewarded trials in terms of deviation, neither for Experiment 1, nor for Experiment 2. If the performance difference between rewarded and unrewarded trials was caused by improved distractor suppression, we would have expected stronger saccadic deviation in trials with a reward. Even if our results cannot ultimately distinguish whether reward facilitates target processing or whether it improves distractor suppression, two further observations from the main experiment support the latter: First, most errors were erroneous responses to the distractor (Figs. 4 and 5) and thus accuracy was mainly determined by successfully avoiding the distractor. The proportion of erroneous responses to the distractor was least in rewarded trials compared with any other condition (Figs. 4 and 5). Second, a performance benefit was observed for latencies within the first 100 ms after target onset (Figs. 3, 4 and 5), which renders it more likely that this performance benefit is related to the distractor (which precedes the target by 187 ms) rather than the target. Our previous findings showed that biases induced by suddenly appearing salient distractors can be overcome 250–300 ms after distractor onset (Wolf & Lappe, 2020, 2021a), which is temporally consistent with the present performance saturation around 100–120 ms after target onset. Taken together, although we cannot ultimately distinguish whether reward aids target facilitation or whether it improves distractor suppression, our results are more consistent with the latter.

We analyzed accuracy and occurrence of specific errors as a function of response time (Figs. 4 and 5). We found higher accuracy in rewarded trials within the first 100 ms after target onset, and a lowered accuracy with a perceptual task for responses initiated between 201 and 255 ms. Most errors were caused by premature responses to the distractor rather than an active gambling behavior (Figs. 4 and 5) and higher accuracy was reflected in a lower number of erroneous responses to the distractor in the respective time windows. However, the lacking difference in strategic errors between relevant and irrelevant trials might be caused by the few errors and the few trials in the time window where these errors mostly occurred.

The common analysis of mean response time and mean accuracy on the one hand and these time courses on the other hand indicates whether changes in behavior are caused by a trade-off between speed and accuracy or by processes that operate outside this trade-off. Theoretically, if two conditions differed due to a traditional speed–accuracy trade-off, this would result in different mean response times and accuracies without a change in the time courses of these two conditions. Indeed, if changes in performance are time-locked to stimulus onset, then the underlying time course should be the same. Thus, changes in speed and accuracy would only result from the way that this time course is sampled. For example, imagine overlapping time courses (as in Fig. 3c) with one reaction time distribution (top panel in Fig. 3c) centered at a time point where performance is already saturated, but the other distribution centered at a time when performance is still poor. This would result in different mean response times and accuracies despite the same underlying time course. Contrary to that, differences in the time courses reflect processes beyond the speed–accuracy trade-off. For example, a shifted time course might be indicative of reduced internal noise that might result in better distractor suppression or facilitated target processing.

Yet, even if the two time courses differ, performance might still be prone to a trade-off between speed and accuracy. We believe that this can account for our results in the reward condition: At large, our results in the reward condition are consistent with the findings obtained by Manohar et al. (2015). We observed that the prospect of reward resulted in more accurate responses shortly after target onset. However, this did not show on the aggregated level (mean accuracies, i.e., averaged across response times). We believe that this is due to two effects cancelling each other out. The first is an earlier saturation of accuracy in rewarded compared with unrewarded trials (Fig. 3a). This first effect would yield better performance in rewarded compared with unrewarded trials. The second effect assumes that behavior in the reward condition is still prone to the classical trade-off between speed and accuracy. Response accuracy increased steadily and saturated at a time point where most responses had not yet occurred. In turn, this also implies that most responses occurred at a time point without a benefit for rewarded over unrewarded trials. Thus, the increased speed in rewarded trials led to a higher fraction of trials with response times at which performance was not yet saturated. Whether the performance benefit in rewarded trials can be observed on the aggregated level will thus also depend on the fraction of trials that occur in the time window where performance is enhanced. This might have been the case if we had decided to use a shorter delay between distractor and target, for example a time between 40 and 120 ms as in Manohar et al. (2015).

In this line of thought, mean response times and accuracies in the task-relevance condition indicated a typical speed–accuracy trade-off: faster, yet less accurate selection in relevant compared with irrelevant trials. This was reflected in a lowered decision threshold in the diffusion model. Thus, participants were less cautious so that they may see the perceptual target earlier, accepting potential errors. This pattern did not only result from the way that the same underlying time course was sampled, because accuracy time courses differed between relevant and irrelevant trials (Fig. 3b). The time course in relevant trials showed a late dip in performance after the initial distractor suppression had already been saturated. Such lapses cannot be attributed to a strategic task avoidance to reduce the overall experimental duration: If participants selected the wrong target, they still had to perform the perceptual task, making it impossible to speed up the experiment by strategically selecting a disc other than the target. Instead, these lapses might be indicative of a diminished task engagement and a lack in motivation to perform well in the task.

Postsaccadic vision of an intrinsically relevant face stimulus did neither affect response speed nor accuracy. In this condition, we used faces of famous people to enable person recognition. Although participants might not have been familiar with all faces, recognition should have been possible in the majority of trials. Hence, our data suggest that neither postsaccadic vision of an intrinsically relevant face stimulus, nor the possibility to recognize a face have the motivational ability to affect oculomotor target selection. Given that targets had only been shown after the saccade, results are essentially unaffected by low-level properties of a peripheral target that have been shown to affect eye movement responses towards faces (Crouzet & Thorpe, 2011; Honey et al., 2008). Even short peripheral glimpses of the target might suffice to induce faster saccades towards faces, even if the saccade is carried out at a later point in time (Xu-Wilson et al., 2009).

We complemented our analysis with a drift-diffusion modelling approach which showed that motivation by reward affected information uptake (i.e., drift rate) and decision threshold (i.e., boundary separation), whereas task-relevance only affected the latter and image content affected neither (Fig. 6). According to the selective influence assumption, instructing participants to either emphasize speed or accuracy should only affect decision thresholds, whereas changing the difficulty of the task should exclusively affect information uptake. Under this assumption, our results are consistent with the conclusion that (i) reward and task-relevance affect the speed–accuracy trade-off, whereas image content does not. Additionally, (ii) motivation by reward increases the amount of information per time, effectively making the task easier when anticipating a reward. This is consistent with the notion that motivation by reward can decrease noise (Manohar et al., 2015). However, this selective influence assumption has recently been challenged (Dutilh et al., 2019; Rae et al., 2014; Rafiei & Rahnev, 2021)—for example, because accuracy instructions that should have exclusively affected the boundary separation parameter additionally affected drift rates or nondecision times. Our present results show that the same pattern that was observed in the boundary separation parameter could also be found in nondecision times. Nondecision times are thought to reflect that part of the reaction time that is not devoted to the decision processes but to other processes such as the motor response or stimulus encoding. Our measure of reaction time, saccade latencies, does not include the time dedicated to the actual movement and there is no reason to assume any difference in the encoding time of the presaccadic display. Hence, this result pattern shows that nondecision times covaried with changes in decision thresholds which appears inconsistent with the selective influence assumption. However, we did not explicitly instruct participants to either emphasize speed or accuracy, but we manipulated the consequences following an accurate response to test whether participants implicitly adjust their trade-off in speed and accuracy. Thus, participants were free to adjust their behavior in any way and we cannot distinguish whether our results are inconsistent with the selective influence assumption or not. In any case, drift-diffusion modelling revealed differences in the underlying decision processes for reward, task-relevance, and intrinsically relevant images.

To conclude, although earlier eye movement responses or a stronger maintenance of saccadic accuracy can be found with monetary reward, perceptual tasks as well as image content, we here show that these visual consequences differ in terms of their motivational abilities. Thus, although these consequences might apparently evoke the same behavior, this is not for the same reason.
